# Interactions between large‐scale and local factors influence seed predation rates and seed loss

**DOI:** 10.1002/ece3.10208

**Published:** 2023-06-28

**Authors:** Eduardo S. Calixto, John L. Maron, Philip G. Hahn

**Affiliations:** ^1^ Entomology and Nematology Department University of Florida Gainesville Florida USA; ^2^ Division of Biological Sciences University of Montana Missoula Montana USA

**Keywords:** density dependence, plant fecundity, resource availability hypothesis, resource concentration, resource dilution, seed predators

## Abstract

Herbivores often have highly variable impacts on plant fecundity. The relative contribution of different environmental factors operating at varying spatial scales in affecting this variability is often unclear. We examined how density‐dependent seed predation at local scales and regional differences in primary productivity are associated with variation in the magnitude of pre‐dispersal seed predation on *Monarda fistulosa* (Lamiaceae). Within *M. fistulosa* populations growing in a low‐productivity region (LPR), Montana, USA, and a high‐productivity region (HPR), Wisconsin, USA, we quantified the magnitude of pre‐dispersal seed predation among individual plants differing in seed head densities. Out of a total of 303 *M. fistulosa* plants that were surveyed, we found half as many herbivores in seed heads in the LPR (*n* = 133 herbivores) compared to the HPR (*n* = 316). In the LPR, 30% of the seed heads were damaged in plants with low seed head density, while 61% of seed heads were damaged in plants with high seed head density. Seed head damage was consistently high in the HPR (about 49% across the range of seed head density) compared to the LPR (45% across a range of seed head density). However, the proportion of seeds per seed head that were destroyed by herbivores was nearly two times higher (~38% loss) in the LPR compared to HPR (22% loss). Considering the combined effects of probability of damage and seed loss per seed head, the proportion seed loss per plant was consistently higher in the HPR regardless of seed head density. Nevertheless, because of greater seed head production, the total number of viable seeds produced per plant was higher in HPR and high‐density plants, despite being exposed to greater herbivore pressure. These findings show how large‐scale factors can interact with local‐scale factors to influence how strongly herbivores suppress plant fecundity.

## Graphical abstract

Herbivores often have highly variable impacts on plant fecundity. We examined how density‐dependent seed predation at local scales and regional differences in community productivity are associated with variation in the magnitude of pre‐dispersal seed predation on *Monarda fistulosa*. Our findings show how large‐scale factors can interact with local‐scale factors to influence how strongly herbivores suppress plant fecundity.

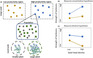

## 1 INTRODUCTION

Variation in the amount of food resources can influence the extent to which those resources are affected by top‐down predation (Hunter & Price, 1992; Power, 1992). This has been particularly well documented for plant‐herbivore interactions (Connell, 1971; Janzen, 1970; Maron et al., 2014; Root, 1973), where the quantity of host‐plant resources can influence how strongly herbivores reduce plant fecundity (Ågren et al., 2008; Comita et al., 2014; Janzen, 1971; von Euler et al., 2014). Multiple hypotheses predict how abiotic variation, and its influences on food resources for herbivores, should affect the magnitude of damage plants suffer from herbivory. At large spatial scales, climate, and underlying soil conditions drive variation in overall primary productivity, i.e., the generation of plant biomass in the ecosystem (Rosenzweig, 1968; Sala et al., 1988). Higher productivity communities are predicted to support greater herbivore abundance, which may inflict greater plant damage than in low productivity communities (Chase et al., 2000; Pennings et al., 2009). These effects can occur across continuous gradients in plant productivity (e.g., Croy et al., 2022; Hahn et al., 2019; Moreira et al., 2018) as well as across populations that occur in discrete high and low productivity habitats (Baskett et al., 2020; Hahn et al., 2021; Robinson & Strauss, 2018).

At small spatial scales, the resource concentration hypothesis (Root, 1973) predicts that insect herbivory should increase with the density of local food resources (e.g., leaves, flowers, fruits, and seeds), because denser plant patches are more easily discovered than low‐density patches, and can attract greater numbers of specialist herbivores (Andersson et al., 2013; Hambäck et al., 2014; Otway et al., 2005). This has been supported by numerous studies in a variety of systems (e.g., Andersson et al., 2013; Barbosa et al., 2009; Hambäck et al., 2014; Otway et al., 2005; Underwood et al., 2014), although there can be considerable variability in how strongly herbivore damage increases with resource density as well as the resulting impact on plant fecundity (Fedriani et al., 2015; Jones & Comita, 2010; Otway et al., 2005; Underwood et al., 2014).

**FIGURE 1:**
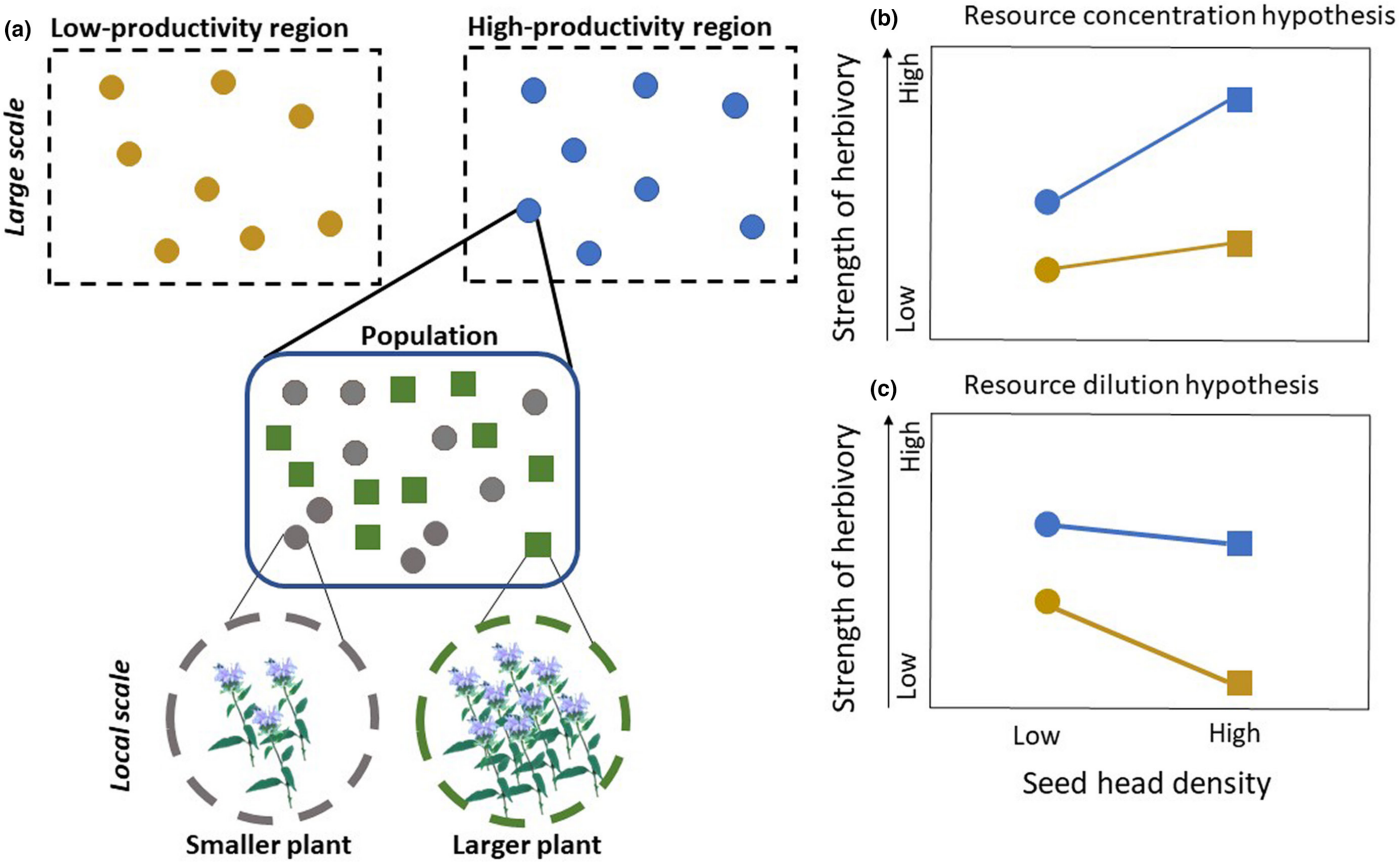
(a) Research design evaluating how multiscale processes influence how strongly herbivores suppress plant fecundity. (b, c) Hypothetical scenarios for the strength of the effects of seed predators across seed head densities per plant at the local scale and regional productivity at the larger spatial scale. It is expected that herbivore pressure is greater in high versus low productivity regions, but the food resource density at local scale may concentrate (b) or dilute (c) the effects imposed by herbivores.

Variation in productivity at larger scales can potentially interact with variation in the density of host plant resources locally to influence the amount of herbivory (Figure 1a), although this has seldom been explored. For instance, in high‐productivity regions (hereafter HPRs), larger plants produce high densities of plant tissue that may accrue greater herbivory than similarly sized individuals in low‐productivity regions (hereafter LPRs) because herbivores are generally more abundant in HPRs versus LPRs (Figure 1b; Hahn et al., 2019; Pennings et al., 2009; Salazar & Marquis, 2012). Alternatively, in LPRs, high‐density patches of plant tissue may experience reduced herbivory compared to HPRs, because herbivore numbers are generally lower in LPRs (Figure 1c). Herbivory “dilution” occurs when denser patches or larger individual plants (with greater numbers of leaves, flowers, fruiting stems, seeds, etc.) suffer less herbivory on a per capita basis than smaller patches or plants (Otway et al., 2005; Stephens & Myers, 2012; Underwood & Halpern, 2012; Xiao et al., 2017). Thus, factors related to overall herbivore abundance, and then their attraction within a population to individual plants, potentially influence how large‐scale and local factors together impact amounts of herbivory.

Here, we assess how a specialist pre‐dispersal seed predator, *Cochylis bucera*, impacts reproductive output (i.e., fecundity) of a widely distributed grassland/prairie forb, *Monarda fistulosa*, across large and small scales. At a large geographic scale, we focus on how seed loss due to herbivory varies between a drier region with cooler growing seasons and therefore lower overall primary productivity, and a wetter region, with warmer growing seasons and higher overall productivity (Hahn et al., 2021). We focus on large‐scale variation in plant productivity (particularly as it relates to above‐ground standing biomass) between regions because it can influence both the size of individual *Monarda* plants as well as herbivore numbers, and therefore can strongly influence plant‐herbivore interactions. Locally, we examined how seed loss varies across *Monarda* plants where flowering stem density can be quite variable (Figure 1a). In single populations, individual *Monarda* plants can produce relatively few flowering ramets (with low seed head density) or a high density of flowering ramets (with high seed head density). Thus, there can be substantial small‐ and large‐scale variations in the density of seed heads available to pre‐dispersal seed predators (Hahn et al., 2021; Keefover‐Ring, 2015).

Regional variation in productivity and local seed head density may interact in several ways to influence how strongly pre‐dispersal seed predation affects plant fecundity (Figure 1b,c). At large spatial scales, we predicted that insect abundance and seed head damage should be greater in the HPR compared to the LPR. At local scales, herbivore abundance and damage could either increase (i.e., resource concentration hypothesis) or decrease (i.e., resource dilution hypothesis) with increasing seed head density. We predicted that the effects of resource concentration on herbivore damage should be stronger in the HPR due to greater insect abundance (Figure 1b), whereas resource dilution should be stronger in the LPR due to lower insect abundance (Figure 1c). To test for these interactive effects, we quantified the number of seed head predators, the probability of seed head damage, and the proportion seed loss due to pre‐dispersal seed predation across multiple *Monarda* populations located in two regions with different productivity. A concentration effect occurs (Figure 1b) when the number of seed head predators, the probability of seed head damage, or the proportion seed loss increase from low to high seed head densities. A dilution effect occurs (Figure 1c) when these herbivore effects (herbivore number, probability of damage, and seed loss) decrease from low to high seed head densities. Dilution effects can also occur if larger plants are better able to attenuate damage, for example, by producing larger seed heads with more seeds per head. Finally, we evaluated how the combined effects of the probability of damage and seed loss per head translate into average seed production at the plant level.

## 2 MATERIALS AND METHODS

### 2.1 Plant and insect natural history

*Monarda fistulosa* (Lamiaceae), also known as wild bergamot or bee balm, is a widely distributed herbaceous perennial forb that inhabits grasslands, prairies, and forest edges across North America. Plants flower in July and August across most of its range. Flowers within a capitulum open centrifugally and are bee‐pollinated (Cruden et al., 1984). Seeds are produced in floral tubes, with four ovules per tube; if one floral tube is fertilized, four seeds are produced, although usually not all tubes in a flower are fertilized (Cariveau & Norton, 2009).

*Monarda* seed heads in both regions are commonly attacked by larvae of at least two species of microlepidoptera: *Pyrausta signatalis* (Family Crambidaeand) and *Cochylis bucera* (Family: Tortricidae). *Pyrausta signatalis*, which was rare in our study, is a specialist herbivore that attacks different species of *Monarda*, consuming mainly reproductive parts, such as flowers and seeds. We found *C. bucera* only inside of seed heads, and since larvae of this species do not have a developed locomotor apparatus, they cannot easily move among seed heads (ESC pers. obs.). Larvae of *C. bucera* have been found to feed on four species in the Lamiaceae family, namely *Monarda fistulosa*, *M. punctata*, *Blephilia hirsuta*, and *Mesosphaerum rugosum* (Santa‐Rita et al., 2022). Of these potential host plants, only *M. fistulosa* occurred at our study sites. Adults are on wing from June to September, and larvae occur during *Monarda* seed production (Davis et al., 1987; Hahn et al., 2021; ESC and PGH pers. obs.), which occurs from August to September in both regions. Identification of *C. bucera* larvae (two larvae from Montana and one larva from Wisconsin) was confirmed by DNA barcoding using the COI primer with standardized protocols (Folmer et al., 1994) and comparing the sequences in the BLAST database (https://blast.ncbi.nlm.nih.gov/Blast.cgi).

### 2.2 Study system

Fieldwork was performed in August 2021 at multiple grassland populations located within two distinct regions (Appendix S1: Table S1), western Montana (LPR) and southern Wisconsin (HPR), USA. In the LPR, *Monarda* populations grow in dry perennial grasslands in the intermountain west, which are dominated by native bunchgrasses (e.g., *Festuca campestris* and *Pseudoroegneria spicata*) and native perennial forbs (e.g., *Achillea millefolium*, *Erigeron pumillus*, *Lupinus serritorium*, and other wildflowers). Most co‐occurring wildflowers bloom in June, which is earlier than *Monarda* in intermountain grasslands. In the HPR, *Monarda* populations grow within tallgrass prairie remnants and old field habitats, which are dominated by native (e.g., *Andropogon gerardii*, *Schizachyrium scoparium*) and non‐native grasses (e.g., *Bromus inermis*, *Poa pretensis*), co‐blooming native and non‐native perennial forbs (e.g., *Coreopsis palmata*, *Dacus carota*, *Dalea purpurea*, *Penstemon digitalis*, *Rudbeckia hirta*), as well as other species that bloom earlier or later than *Monarda* (e.g., *Asclepias*, asters, *Solidago*). In the LPR in western Montana, summer temperature (mean 17.0 ± SD 1.2°C) and annual precipitation (mean 384.4 ± SD 37.1 mm) are 1.2 and 2.2 times lower (Figure S1) than at sites in southern Wisconsin (HPR; mean summer temperature = 20.4 ± SD 0.32°C; summer rainfall = mean 836.8 ± SD 24.6 mm; Figure S1). These climatic conditions are associated with differences in plant size (height and number of seed heads), where plant height and productivity are substantially lower in the LPR compared to the HPR (Hahn et al., 2021). Soil properties, such as cation exchange capacity, soil nitrogen, and percent organic matter, are generally similar between regions, with the exception of soil phosphorous which is higher in Montana than Wisconsin (Hahn et al., 2021).

Within each region, we studied seven (LPR) or eight (HPR) spatially separated replicate populations of *Monarda* (*n* = 15 populations total; Table S1). In the LPR, our study populations were 0.5–99 km apart (mean = 41 ± SD 30 km); the two closest populations (0.5 km apart) were situated on opposite slopes of a mountain. In the HPR, populations were 16–160 km apart (mean = 86 ± SD 42 km; Table S1). In each study population, we selected 20–25 plant individuals of *Monarda*, which varied in seed head density, the resource that is important to pre‐dispersal seed predators. Although the number of seed heads per plant is likely a function of plant age and microsite conditions, we were not able to measure these factors in our study. Given that *Monarda* grows clonally (Keefover‐Ring, 2015), we counted all seed heads within a 0.25 m^2^‐circular plot and treated each plot as a plant individual. Plants within the same population were at least 3 m apart. Based on our field observations and experiments in common gardens in both regions (Hahn et al., 2021 and ESC pers. obs.), ramets of the same plant often occur close to each other and almost always within 0.25 m^2^. Thus, each plot likely encompasses one individual plant (see Hahn et al., 2021; Keefover‐Ring, 2015, 2022). Within each population, plants were classified as low‐density (less than 10 seed heads per plant; 10–15 plants per population) or high‐density (10–60 seed heads per plant; ~10 plants per population). *Monarda* plants in the LPR had on average 3.8 ± 1.4 (mean ± standard deviation) and 19.5 ± 5 seed heads in low‐ and high‐density plants, respectively. *Monarda* plants in the HPR had on average 5.6 ± 3.7 and 22.8 ± 8.2 seed heads in low‐ and high‐density plants, respectively.

### 2.3 Seed head collection

Within each *Monarda* plant, we counted the total number of seed heads, and collected seed heads for determination of seed production and loss due to seed predation. For plants with more than five seed heads, we selected the five tallest heads to harvest to standardize collection across populations. In plants with five or less seed heads, we collected all heads. Seed heads were collected after seeds had matured but prior to any seed dispersal. Seed heads from the same plant stored together in the same coin envelope were brought to the laboratory at the University of Florida, Gainesville, USA, where they were processed. For each seed head, we measured its diameter, counted the total number of seeds produced, and recorded the presence of insects and damage.

### 2.4 Data analysis

All analyses were conducted in R version 4.2.2 (R Core Team, 2021). Models were fit using “glmmTMB” (Brooks et al., 2017), residuals were assessed using DHARMa (Hartig, 2020) and Wald *χ* ^2^ and *p*‐values were obtained by using the Anova() function from package “car” (Fox & Weisberg, 2019). We used the emmeans() function from package “emmeans” (Lenth, 2020) to obtain estimated marginal means, which were back‐transformed to the original scale.

We determined how regional productivity and seed head density per plant independently and interactively influence herbivore pressure and consequently seed loss. We measured three metrics, the number of seed predators per head, the probability of seed head damage, and the proportion of seed loss. In these analyses, the region is represented by multiple (*n* = 7–8) populations, but we recognize region is not truly replicated. For all metrics, we fit generalized linear mixed‐effect models (GLMMs). To test whether denser plants have a higher (Figure 1b) or lower (Figure 1c) number of seed head predators per head and whether this effect was stronger in the HPR than LPR, we fit the number of seed head predators as the response variable, region, seed head density per plant (low or high), and the interaction of region and seed head density as fixed effects. We used the negative binomial distribution, which controlled for overdispersion in the data. Plant ID nested within a population nested within a region was fit as a random effect in our model.

To test whether denser plants have a higher (Figure 1b) or lower (Figure 1c) probability of seed head damage and whether this effect was stronger in the HPR than LPR, we fit a model similar to the one used to analyze the number of seed predators per head. However, we now fit the presence or absence of seed head damage as the response variable using a binomial distribution.

To analyze seed loss at the seed head level, we first estimated seed loss by using both undamaged and damaged seed heads because seed predators consume seeds completely, making it impossible to quantify seed loss directly from damaged seed heads. To do this, we fit the total number of seeds per head as the response variable with the interaction of region, seed head density per plant, and the presence or absence of damage as fixed effects. In this model, we also added seed head diameter as a covariate, which was necessary to control for differences in seed head size among individuals within each population when estimating seed loss (Figure S2). Plant ID nested within a population nested within a region was included as a random effect in our model. Finally, we calculated the proportion seed loss using post hoc contrasts (*emmeans* package) comparing seeds produced on average in damaged heads divided by undamaged heads for different treatment combinations (proportion seed loss = 1−(seed number in damaged heads/seed number in undamaged heads)).

To evaluate how seed predation at the seed head level translates into seed production at the plant level, we combined effects of the probability of damage and seed loss per head. Because we could not robustly estimate seed loss on an individual damaged seed head (because of the issues described above), we used the estimates of our models to estimate total seed production per plant for the combinations of the two density treatments and regions. We estimated seed loss per plant by quantifying the ratio between the total number of seeds produced per plant with the presence of damage and the total number of seeds per plant if there was no damage.

## 3 RESULTS

### 3.1 Abundance of pre‐dispersal seed predator

**FIGURE 2:**
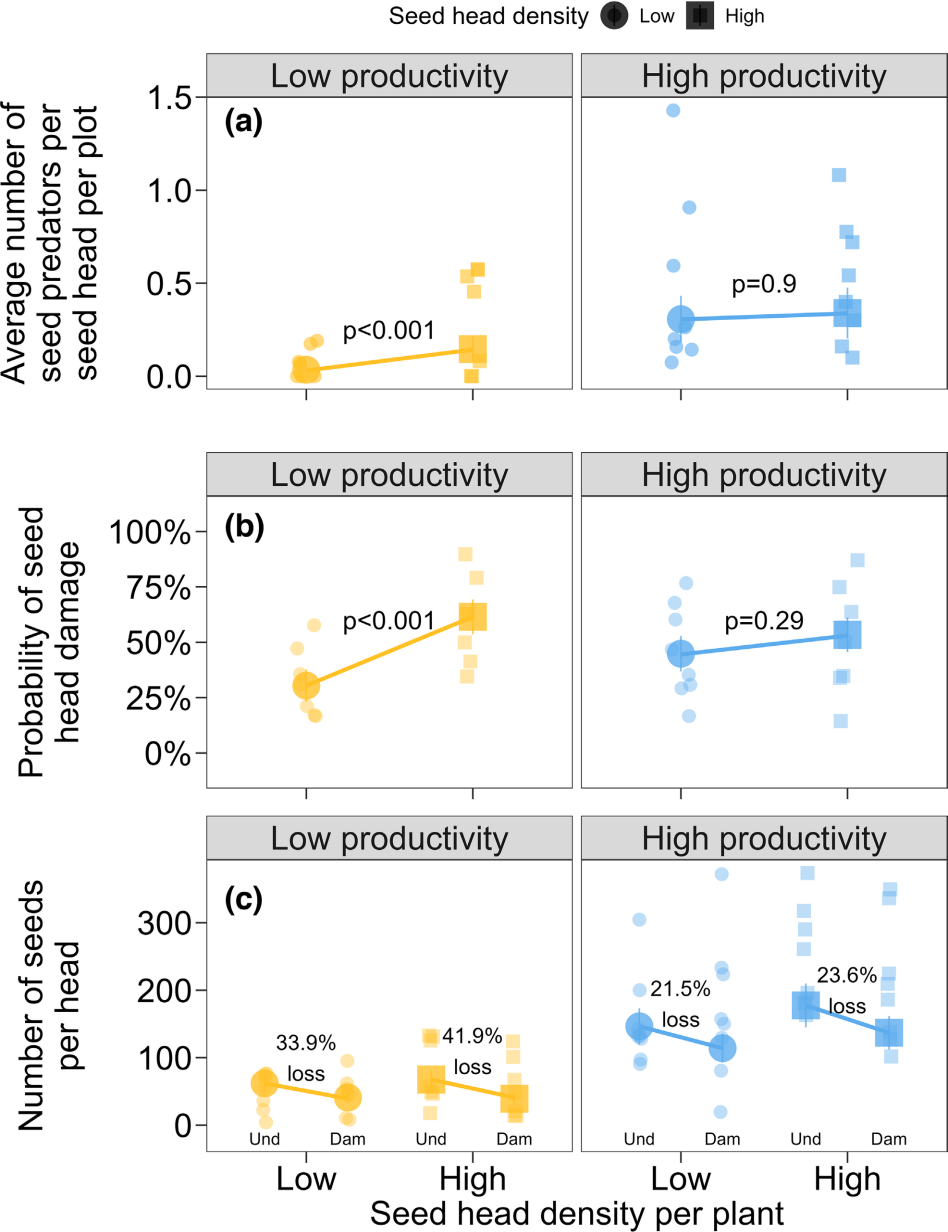
Influence of seed head density on (a) number of seed predators, (b) probability of seed head damage, and (c) the proportion of seed loss, represented by the variation in seed production between undamaged and damaged seed heads across regional productivity. In (a) and (b) “regional productivity × seed head density” interaction term was significant (*p* < .001). Large symbols represent the mean (±SE) values per region per group of seed head density (low density—circle, high density—square). Small dots represent the mean values per region per population per group of seed head density in (a) and (b), and mean values per region per population per group of seed head density in damaged and undamaged seed heads in (c). Data are back‐transformed to the original scale for plotting.

From a total of 1224 seed heads harvested from the 303 plants surveyed, 287 (23.4%) heads had one or more *C. bucera* larvae present and six individuals of *P. signatalis* (two in the LPR and four in the HPR). In total, we found 449 individuals of *C. bucera* (20 in low‐density plants and 113 in high‐density plants in LPRs, 124 in low‐density plants, and 192 in high‐density plants in HPRs). Most of the seed heads containing this seed predator had only one individual (79.4% of the flower heads in LPRs, and 59.6% in HPRs), although we found up to seven individuals in the same head. The number of seed predators per seed head varied with seed head density per plant (*χ* ^2^ = 10.5, *p* = .001), and seed head density × region interaction (*χ* ^2^ = 19.6, *p* < .001, Figure 2a). Region had a marginal effect on the number of *C. bucera* seed predators per seed head (*χ* ^2^ = 3.5, *p* = .059). The average number of seed predators per seed head was similar across seed head densities in the HPR (about 0.32 seed head predator averaged across seed head density treatments; contrast between low‐ and high‐density plants: *t* = 0.68, *p* = .9, Figure 2a). In the LPR, the average number of seed predators per seed head varied from 0.03 (SE 0.01) at low density to 0.14 (SE 0.06) at high density (contrast: *t* = 5.4, *p* < .001, Figure 2a). Since we found only six individuals of the seed predator *P. signatalis* in *Monarda* seed heads, we focus hereafter on *C. bucera*.

### 3.2 Probability of seed head damage

Seed head damage was common, with 596 (49%) seed heads damaged of the total harvested (1224). In about half of the cases (52.3% of damaged heads), we found evidence of damage without finding an insect. The probability of seed head damage varied with local seed head density (*χ* ^2^ = 33.6, *p* < .001), but not with region (*χ* ^2^ = 0.01, *p* = .89). However, there was a significant seed head density × region interaction (*χ* ^2^ = 11.7, *p* < .001, Figure 2b). The probability of seed head damage was consistently high in the HPRs (about 49% probability of damage averaged across seed head density treatments; contrast between low‐ and high‐density plants: *t* = 1.75, *p* = .29, Figure 2b). In the LPR, the probability of seed head damage varied from 30% (SE 7%) at low density to 61% (SE 7%) at high density (contrast: *t* = 6.5, *p* < .001, Figure 2b).

### 3.3 Seed loss

At the seed head level, we compared the total number of seeds produced between undamaged and damaged seed heads across seed head densities and regions. The total number of seeds produced per head was significantly influenced by region, seed head density per plant, presence of damage in seed heads, the interaction between region and damage, and seed head diameter (Figure 2c; Table 1; Figure S2). Other interactions between variables were not significant (Table 1). Seed heads in HPRs produced on average 3.5 times more seeds (mean 142 seeds ±24 SE) than seed heads in LPRs (mean 51 seeds ±9 SE; Figure 2c). In addition, when attacked by pre‐dispersal seed predators (seeds produced in damaged divided by undamaged seed heads), seed heads in LPR proportionally lose twice as many seeds (37.9% on average ± 6% SE across densities of seeds heads) than in HPR (22.5% on average ± 2% SE across densities of seed heads; Figure 2c). Finally, seed head size positively influenced the number of seeds produced per head (β_(slope)_ = 0.13 ± 0.008 SE, Table 1, Figure S2).

**TABLE 1:** Generalized Linear Mixed‐Effect Model results of the effects of regional productivity (Region), density of seed heads per plant (Density), presence or absence of damage (Damage), the interactions among these variables, and the seed head diameter as a covariate on the total number of seeds produced per head.

Predictors	Wald *χ* ^2^	*p*
Region	16.7	**.001**
Density	5.6	**.017**
Damage	55.5	**.001**
Seed head diameter	252.8	**.001**
Region: density	3.07	.079
Region: damage	6.07	**.013**
Density: damage	0.5	.47
Region: density: damage	0.41	.51

*Note*: Significant values are in bold.

Combining the information above to estimate seed production at the plant level, the total number of seeds produced is higher in the HPR than in LPR, and in high‐ versus low‐density plants (Table 2). We estimated a total of 2098 seeds produced in high‐density plants in HPR, which was 4.6, 3.6, and 14.7 times higher than low‐density plants in HPR, high‐density plants in LPR, and low‐density plants in LPR, respectively (Table 2). Although there was greater absolute production of seeds per plant in the HPR and in high‐density plants, we found that the proportion seed loss was high and constant across seed head densities in the HPR (Table 2). High‐ and low‐density plots showed a proportion seed loss of 39.5% and 36.4%, respectively. In LPR, the proportion of seed loss per plot increased from 14.8% in low‐density plots to 31.8% in high‐density plots (Table 2), showing a resource concentration effect.

**TABLE 2:** Estimated proportion seed loss per plant between combinations of seed head density and productivity regions based on the models predicting the probability of seed head damage and seed loss per head.

Region	Seed head density	Mean number of seed heads per plant	Estimated total number of seeds per plant without damage	Estimated total number of seeds per plant with damage	Estimated seed loss per plant
LPR	Low	3.7	166.8	142.2	14.8%
LPR	High	18.6	851.8	580.9	31.8%
HPR	Low	5.6	709.5	451.1	36.4%
HPR	High	22.8	3470.1	2098.6	39.5%

## 4 DISCUSSION

Understanding the drivers of spatial variability in herbivory has challenged ecologists for decades (Chamberlain et al., 2014; Maron et al., 2014). In our study, we assessed the attack rates of a specialist pre‐dispersal seed predator and resultant seed loss at different spatial scales. We found that the local‐scale effect of seed head density interacts with the large‐scale effect of regional productivity influencing the number of seed predators per head, the probability of seed head damage, and the proportion seed loss per seed head. Within the low‐productivity region (LPR), the number of seed predators per head and the probability of damage increased with seed head density per plant, as predicted by the resource concentration hypothesis (Root, 1973), whereas herbivore number and damage were consistently high in the high‐productivity region (HPR; Figure 2). When attacked by a seed predator, the proportion seed loss per head was similar between high‐ and low‐density plants within each region but was about 2‐fold stronger (~38% loss in both low‐ and high‐density plants) in the LPR compared to HPR (22% loss on average). A likely explanation for these findings is that variation in seed production per head influenced seed loss. Plants with larger seed heads produced more seeds per head (Table 1, Figure S2), and ultimately experienced lower seed loss. While the proportion seed loss per plant was consistently high in HPR, ultimately plants produced a greater number of viable seeds per plot (Table 2). Thus, although herbivore pressure was consistently high in the HPR (threefold the average number of seed predators per head than LPR and ~49% heads attacked), the larger size of the seed heads and the greater number of seed heads per plant diluted the impacts caused by herbivores through high levels of seed output in the more productive region (Table 2, Figure S2). These results contribute toward a better understanding of how multi‐scale factors can influence pre‐dispersal seed predation and the consequences for plant fecundity.

By examining multiple metrics of damage, from the probability of attack to seed loss per head and per plant, our study provides insight into how herbivores respond to host plant resource density (i.e., number of seed heads per plant and number of seeds produced per plant) at different temporal sequences of attack. First, a herbivore must find its host plant, which can be influenced by resource density (Bell, 1990; Otway et al., 2005; Root, 1973), in our case, the number of seed heads per plant. In the LPR, denser plants (higher number of seed heads) had a higher number of seed predators and higher probability of damage when compared to low‐density plants. These results are consistent with the resource concentration hypothesis (Figure 1b). Since visual and olfactory cues can influence insect searching behavior (Mendes‐Silva et al., 2021) and damaging patterns (Andersson et al., 2013; Bell, 1990; Hambäck & Englund, 2005), plant apparency and the nature of the spatial distribution of resources in LPR and HPR are potential explanations for the variability between regions (Castagneyrol et al., 2013; Hambäck & Englund, 2005). In the LPR, where *Monarda* plants are smaller (Hahn et al., 2021) and tend to be more spatially dispersed (ESC pers. obs.), high density of seed heads might be more apparent to herbivores and thus are colonized at higher rates (Barbosa et al., 2009; Castagneyrol et al., 2013; Hambäck et al., 2014; Underwood et al., 2014; Xiao et al., 2017). In contrast, in the HPR where longer growing seasons and rainfall drive higher plant productivity (Hahn et al., 2021) and likely plant density with individuals more evenly dispersed (ESC pers. obs.), different levels of seed head density are likely equally apparent. Additionally, herbivores were 2.3 times more abundant in the HPR (*n* = 316 total herbivores recovered in seed heads) compared to the LPR (*n* = 133 total herbivores; Figure 2a). Thus, these findings suggest that less dense plants benefit by escaping host detection in LPRs whereas host plant detection seems similar regardless of density in the HPR. Although plant diversity might differ between LRR and HRR, evidence shows that most specialist herbivores are unlikely to be strongly influenced by heterospecific neighbors (Hahn & Cammarano, 2023). In sum, our results suggest that densities of host plant resources (seed heads per plant) for specialist herbivores, potentially in addition to other factors such as the overall population size, spatial distribution, and herbivore abundance are important for predicting herbivory levels.

Once the host plant is found and herbivores start consuming plant tissue, genetic and phenotypic differences among host plants can influence the negative impact of damage on seed output (Hawkes & Sullivan, 2001). We previously found that plants from the LPR were more defended with terpenoids than plants from the HPR when grown in a common garden (Hahn et al., 2021). In this study, we found that the proportion seed loss per head in LPR was actually twofold higher than in HPR (Figure 2c), but estimated proportion seed loss per plant was consistently higher in HPR because of greater herbivore pressure. Yet, overall, the total number of seeds produced per plant was higher in HPR and high‐density plants because these plants produced more seed heads (Table 2). After being damaged, some plants can reallocate energy to produce additional reproductive components, such as flowers and seeds, which can balance or even increase the overall seed output when compared to undamaged plants (Aguirrebengoa et al., 2021; Lortie & Aarssen, 2000). Although this is an important mechanism for compensating for herbivore damage and increasing overall seed output (Strauss & Agrawal, 1999), it is probably minimal in *Monarda* due to the timing of damage. The seed predators seem to do most of the damage late after flowering, during seed development, and once seeds have ripened. Instead, our results suggest that plants in more productive regions and with greater density of seed heads are able to buffer against seed loss by producing a greater number of seeds per head and per plant (Figure 2, Table 2, Figure S2), resulting in a dilution effect despite overall greater levels of damage (Fedriani et al., 2015; Jones & Comita, 2010; Otway et al., 2005; Stephens & Myers, 2012).

One strength of our study design, using multiple replicated populations within two strongly contrasting regions, is that it allowed us to address variation in the strength of herbivory across small as well as larger regional scales. Yet a drawback of our design was that we were not able to replicate “regions”, making it difficult to pinpoint the factors that caused *Monarda* density, herbivore numbers, and, therefore, herbivore damage to vary between regions. However, there is strong evidence that regional primary productivity is positively related to levels of precipitation and temperature (Del Grosso et al., 2008; Sala et al., 1988, 2012). Therefore, the clear differences in rainfall and growing season temperature between regions drive greater overall primary productivity in Wisconsin versus western Montana grasslands due to a longer growing season (Figure S1; Hahn et al., 2021). In turn, this higher productivity, together with climatic variables, translates into higher abundance and levels of damage caused by herbivores (Figure 2; Chase et al., 2000; Hahn et al., 2019; Pennings et al., 2001, 2009). Although it is difficult to generalize our conclusion to other study systems, studies considering continuous gradients (Croy et al., 2022; Hahn et al., 2019; Lehndal & Ågren, 2015) or contrasting environmental conditions (Baskett et al., 2020; Hahn et al., 2021; Robinson & Strauss, 2018) have shown that herbivore impacts are often greater within plant populations growing in high productivity sites versus low productivity sites. Our study adds to this growing knowledge base by demonstrating that the response of herbivores to significant changes in regional productivity can account for seemingly contradictory disparities between damage rates and effects on plant performance at local scales. Continued studies of environmental gradients will allow for synthetic inference of which factors and types of gradient (continuous vs. discrete) most strongly impact the ecology and evolution of plant‐herbivore interactions (Moreira et al., 2018; Robinson & Strauss, 2018).

Herbivores can play an important role in affecting plant reproductive output and abundance (Maron, 1998; Myers & Sarfraz, 2017). To understand this role, studies have attempted to predict impacts of herbivores on plant fecundity at different temporal and spatial scales (Fedriani et al., 2015; Jones & Comita, 2010; Otway et al., 2005; Xiao et al., 2017). Our results suggest that the interaction of local (seed head density per plant) and larger (regional primary productivity) spatial scale factors can affect the strength of the impacts of pre‐dispersal seed predation on plant fecundity. Specifically, we show that counterintuitive differences between damage rates and consequences for plant performance at local scales can be explained by how herbivores and seed production respond to large‐scale differences in regional productivity. Additionally, variation in plant traits (i.e., seed head size, and density of seed heads) also impacted the strength of herbivory on seed loss, where plants with larger seed heads and plants with a higher density of seed heads were better able to buffer the negative effects of herbivory. Our study, therefore, highlights that understanding the larger‐scale context in which local plant‐herbivore interactions play out can importantly predict the consequences of these interactions for plant performance. Future studies investigating herbivore impacts with regard to food resource density would benefit from framing these local interactions within the larger context of environmental productivity.

## AUTHOR CONTRIBUTIONS

**Eduardo S. Calixto:** Conceptualization (equal); formal analysis (lead); methodology (equal); writing – original draft (lead). **John L. Maron:** Conceptualization (equal); funding acquisition (lead); methodology (equal); project administration (lead); resources (lead); supervision (equal); writing – review and editing (equal). **Philip G. Hahn:** Conceptualization (equal); formal analysis (supporting); funding acquisition (lead); methodology (equal); project administration (lead); resources (lead); supervision (lead); writing – review and editing (equal).

## CONFLICT OF INTEREST STATEMENT

The authors have no conflicts of interest to declare.

## Supporting information


Data S1:

## Data Availability

The data that supported the findings of this study are openly available in Dryad repository at https://doi.org/10.5061/dryad.p8cz8w9w7. DNA sequences: Genbank accessions OR139613–OR139615.
