# Supplementary material for: Interactions between large‐scale and local factors influence seed predation rates and seed loss
Source: Ecol Evol. 2023 Jun 28;13(6):e10208. doi: 10.1002/ece3.10208 (PMC10307795; doi:10.1002/ece3.10208)
Supplement: Supplementary file 1 — Data S1: [file ECE3-13-e10208-s001.docx]

**Supporting information**

**Interactions between large-scale and local factors influence seed predation rates and seed loss**

Eduardo S Calixto^1*^, John L Maron^2^, Philip G Hahn^1^

^1^ Entomology and Nematology Department, University of Florida, Gainesville, FL, USA

^2^ Division of Biological Sciences, University of Montana, Missoula, MT, USA

*Corresponding author: ES Calixto, [calixtos.edu@gmail.com](mailto:calixtos.edu@gmail.com)

**Table S1**

Table S1 – Coordinates of the 15 populations of *Monarda fistulosa* plants surveyed. MT – Montana, WI – Wisconsin. LPR – Low-productivity region, HPR – High-productivity region.

| US State | Region | Population code | Latitude | Longitude |
| --- | --- | --- | --- | --- |
| MT | LPR | BF | 46.97046 | -112.965 |
| MT | LPR | CWN | 47.05124 | -113.378 |
| MT | LPR | FF | 46.74837 | -114.177 |
| MT | LPR | MJ | 46.89182 | -113.946 |
| MT | LPR | MS | 46.84029 | -113.979 |
| MT | LPR | MS2 | 46.84107 | -113.981 |
| MT | LPR | NH | 46.8877 | -113.988 |
| WI | HPR | BEN | 42.55894 | -87.9806 |
| WI | HPR | BOL | 43.16316 | -89.5096 |
| WI | HPR | GRE | 43.02718 | -89.4373 |
| WI | HPR | MEQ | 43.19737 | -88.023 |
| WI | HPR | SCH | 42.95387 | -89.8688 |
| WI | HPR | SRD | 42.99123 | -89.0588 |
| WI | HPR | UWM | 43.38838 | -88.0241 |
| WI | HPR | UWW | 43.01665 | -88.4392 |

**Figure S1**


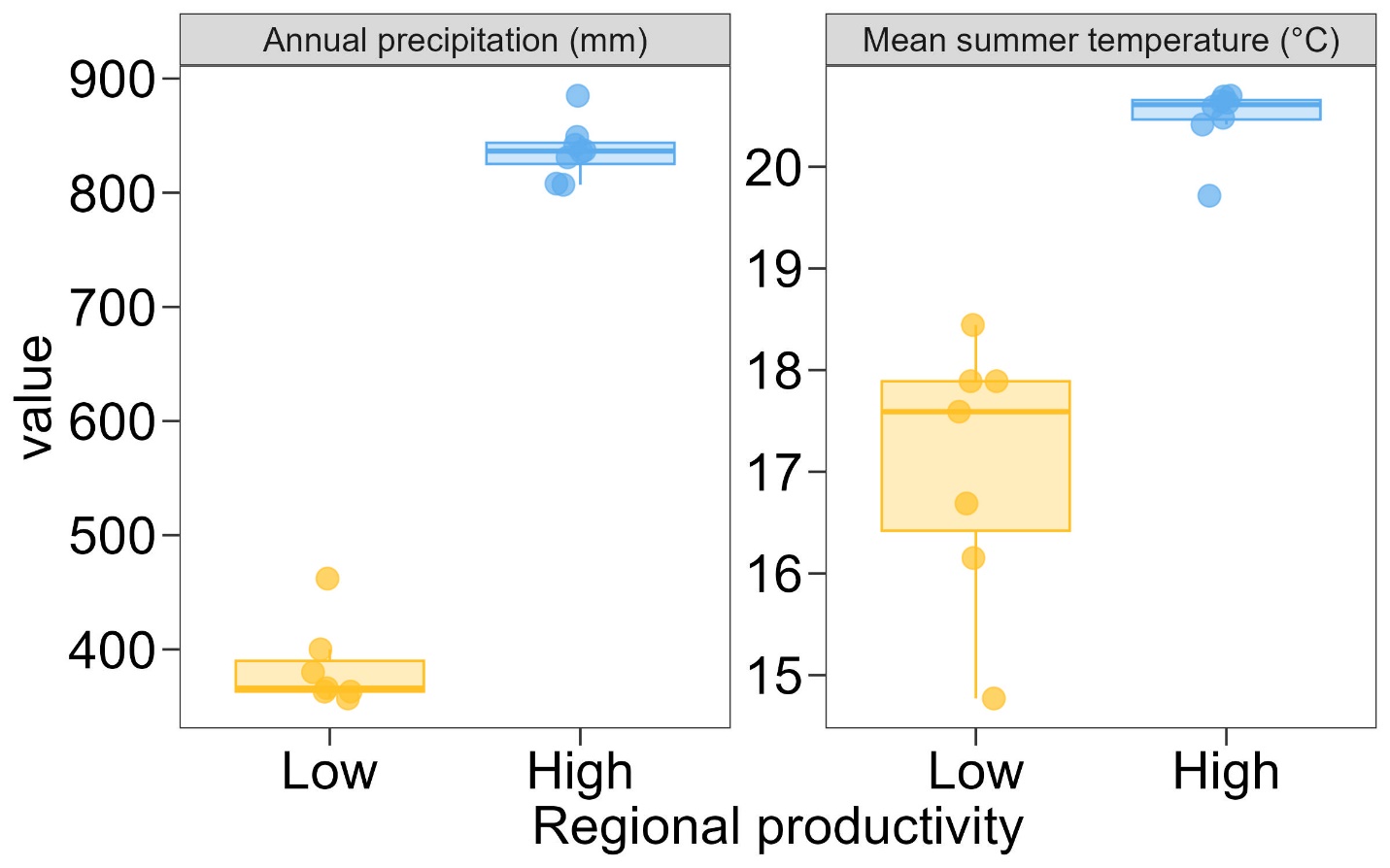


Fig. S1 – Variation in annual precipitation (MT – 384.4 ± 37, WI – 836.8 ± 24; mean ± SD) and mean summer temperature (MT – 17.0 ± 1.2, WI – 20.4 ± 0.3; mean ± SD) between regions of different productivity. Small dots represent the mean values of temperature and precipitation per population. Low-productivity region – Montana, High-productivity region – Wisconsin.

**Figure S2**


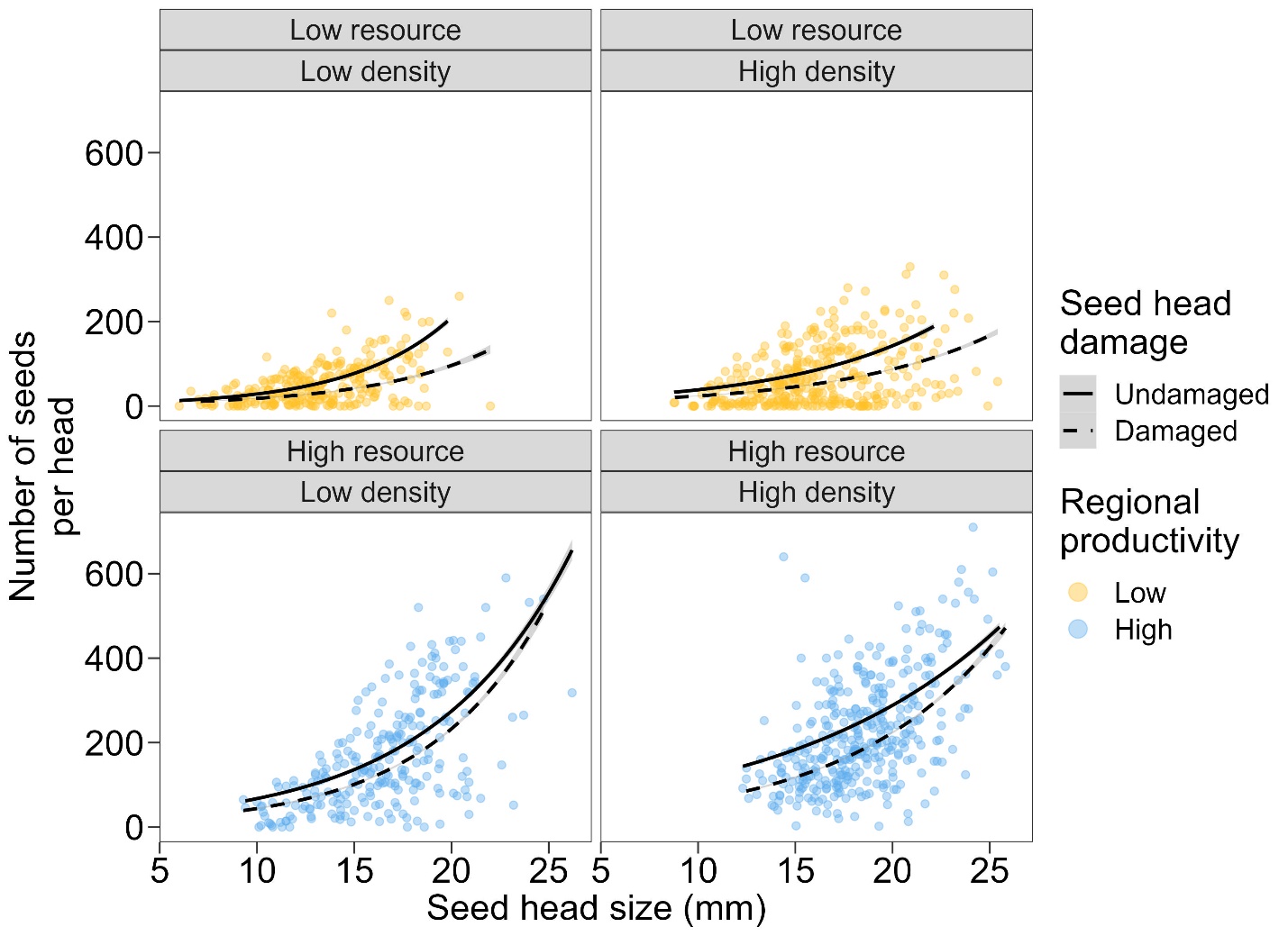


Fig. S2 – Influence of seed head size and seed predator damage on the number of seeds produced per head across different densities of seed heads per plant (low and high density) in regions with different primary productivity (low and high resource). Lines represent the best fit between the predictor and response variable and were fit using a Poisson distribution.
